# Solubilized melanin suppresses macrophage function

**DOI:** 10.1002/2211-5463.12615

**Published:** 2019-03-15

**Authors:** Katsuya Tajima, Daisuke Yamanaka, Ken‐ichi Ishibashi, Yoshiyuki Adachi, Naohito Ohno

**Affiliations:** ^1^ Tokyo University of Pharmacy and Life Sciences Hachioji Japan

**Keywords:** *Aspergillus*, *Cryptococcus*, laccase, L‐DOPA, macrophage, melanin

## Abstract

Melanin‐producing *Cryptococcus* and *Aspergillus* are highly invasive and can suppress or escape the immune system of the host. Since non‐melanin‐producing strains do not affect the immune system, melanin may play a role in immune system suppression. Artificial melanin synthesized using conventional methods is insoluble, making structural and functional analysis of this chemical difficult. In this study, we describe a melanin solubilization method based on polymerization of homogentisic acid (solubilizing component) and an equivalent amount of L‐DOPA in the presence of laccase. In addition, we investigated the effect of melanin on the immune system. Homogentisic acid and L‐DOPA mixed melanin (HALD), the synthetic solubilized melanin, did not exert a cytotoxic effect on mouse macrophages. HALD suppressed cytokine and reactive oxygen species production by macrophages when they were stimulated by fungal components. HALD also suppressed the phagocytosis of fungal components by macrophages. These results suggest that HALD can suppress the function of macrophages without causing cytotoxicity.

**Abbreviations**
BMM: bone marrow macrophage
CSBG: *Candida*‐soluble cell wall β‐d‐glucan
DIW: distilled ion water
GM‐CSF: granulocyte–macrophage colony‐stimulating factor
HA: homogentisic acid
HALD: homogentisic acid and L‐DOPA mixed melanin
IL: interleukin
LD: L‐DOPA
MTT: 3‐(4,5‐dimethylthiazol‐2‐yl)‐2,5‐diphenyl‐tetrazolium bromide
OX‐CA: NaClO‐oxidized *Candida*
PMA/i: phorbol myristate acetate and ionomycin
RLU: relative light unit
ROS: reactive oxygen species
TNF‐α: tumor necrosis factor α
ZYM: zymosan A

*Cryptococcus* is a basidiomycete fungus with a thick capsule, and is widely distributed in the environment, including the air, soil, and plants 1. Cryptococcosis, which results when *Cryptococcus* becomes infectious, is the most common outcome of invasive fungal infections in healthy people 2, 3. *Cryptococcus* mainly infects the lungs and skin, resulting in lesions; however, it is often disseminated to the central nervous system such as the brain, causing disseminated infections such as meningitis 4. Several fungi, including those of the genus *Cryptococcus*, can produce brownish melanin pigments. Melanin is a pigment formed in animals, plants, protozoa, and some fungi. It is a negatively charged pigment of high molecular mass that is composed of polymerized phenolic and/or indolic compounds 5. Melanin can be converted and produced from diphenol and homogentisic acids (HA) 6, 7.

In recent years, genetic studies have demonstrated the pathogenicity of pigments in various microorganisms 4. For example, pyomelanin, a soluble pigment component produced by *Pseudomonas aeruginosa* and *Aeromonas* spp., is involved in the defensive mechanism against antibiotics and oxidative stress 8. Melanin‐producing strains of *Cryptococcus* can suppress the host immune response and inflammatory response 9, 10, and can result in fatal infections in the central nervous system with damage to the brain. However, fatal infections have not been reported for non‐melanin‐producing strains 4, 11. Non‐melanin‐producing strains are less invasive and cannot survive in the spleen, liver, and brain of animals. This may be attributed to the decreased resistance of the non‐melanin‐producing strains to phagocytic cells. Melanin‐producing strains have been demonstrated to interfere with endogenous antimicrobial proteins and exhibit resistance to phagocytic cells 5, 10. Furthermore, melanin‐producing strains have been reported to decrease *Cryptococcus*‐specific lymphocytes by suppressing T‐cell immunity and tumor necrosis factor α (TNF‐α) production by macrophages 7, 12.

Conidia of *Aspergillus* contain melanin, which is a pigment component. It has been found that the binding of complement component 3 (C3) is increased in a mutant strain that is deficient in melanin compared to that in a wild‐type strain 13. It has also been reported in macrophages that when phagosomes are fused with lysosomes containing phagocytized mutant strains that cannot produce melanin, acidification in phagosomes increases 14, 15. Therefore, it is indicated that melanin in *Aspergillus* suppresses the formation of phagolysosome and production of reactive oxygen species (ROS).

Previous findings have indicated that melanin may affect the immune system. Nevertheless, since synthetic melanin produced by the conventional method is completely insoluble 16, melanin has not been reported to have a physiologically active effect on the immune system. A non‐melanin‐producing strain cannot produce melanin since the mRNA expression of laccase is suppressed 17, 18, 19, 20. Therefore, fungi require laccase to produce melanin.

In the mouse model of invasive pulmonary aspergillosis, the pathogenicity of melanin‐deficient strains is found to be decreased 21. In clinical practice, melanin pigment production is considered a major pathogenic factor of *Cryptococcus*; however, the function and role of melanin have not been clarified. To clarify the effect of melanin on the immune system, a solubilized melanin was artificially synthesized, and its effect on the immune system was examined in this study.

## Materials and methods

### Animals

Male C57BL/6 mice were purchased from Japan SLC (Shizuoka, Japan). The mice were housed in a specific pathogen‐free environment and were used at 6–10 weeks of age. All animal experiments were performed in accordance with the guidelines for laboratory animal experiments provided by Tokyo University of Pharmacy and Life Sciences. Each experimental protocol was approved by the Committee for Laboratory Animal Experiments at Tokyo University of Pharmacy and Life Sciences (P18‐35).

### *In vitro* synthesis of solubilized melanin

Solubilized melanin was synthesized using HA and L‐DOPA (LD). The time course of the change in absorbance at 405 nm at room temperature was recorded up to 10 h. The assay mixture contained 100 mm sodium acetate buffer (pH 5.0), 42.5 μg·mL^−1^ laccase, 0.19 mg·mL^−1^ HA, and 0.19 mg·mL^−1^ LD. Laccase inactivation was performed at 100 °C for 20 min.

### Synthesis of the solubilized melanin

The solubilized melanin homogentisic acid and L‐DOPA mixed melanin (HALD) was synthesized by oxidative polymerization of LD (Amano Enzyme Inc., Aichi, Japan) and HA (Tokyo Chemical Industry Co., Ltd, Tokyo, Japan) catalyzed by laccase. Laccase‐catalyzed polymerization was performed as follows: 45 mg of LD and 45 mg of HA were dissolved in 153 mL of Milli‐Q water (Merck KGaA, Darmstadt, Germany) degassed by autoclaving (121 °C, 20 min). The precursors were mixed with 8.5 mg of laccase dissolved in 17 mL of 1 m sodium acetate buffer (pH 5.0) degassed by autoclaving (121 °C, 20 min). The synthesis of HALD was shaded and carried out at a specific oxygen partial pressure, and the reaction rate was controlled. During the reaction, the solution was incubated at room temperature for 24 h in the dark. After completion of the reaction, it was heated at 100 °C for 20 min to inactivate the laccase. After heating, the mixture was extensively dialyzed (molecular mass cut‐off: 50 000 Da) against deionized water for 2 days. Subsequently, the reaction solution was quantified using the Folin–Ciocalteu method (LD conversion) as described in a previous study 22. The samples were dissolved in endotoxin‐free water (1 mg·mL^−1^). The solubilization of HALD was evaluated using an optical microscope. The yield of HALD was determined using the Folin–Ciocalteu method in terms of LD equivalents. To confirm the solubility of melanin, filtration was carried out using a 0.2 μm cellulose acetate membrane filter (Asahi Glass Co., Ltd, Shizuoka, Japan), and the change in color tone of the solution before and after filtration and the particulate matter in the filter were examined. Elemental analysis of each sample was conducted at the Laboratory for Analytical Chemistry, Tokyo University of Pharmacy and Life Sciences.

### Preparation of particulate fungal components

NaClO‐oxidized *Candida* (OX‐CA) was prepared according to a previously described procedure. In brief, acetone‐dried *Candida albicans* cells (2 g) were suspended in 200 mL of 0.1 m NaOH and oxidized with an appropriate volume of NaClO solution for 1 day at 4 °C. After the reaction was completed, the reaction mixture was extensively dialyzed with distilled water to collect the non‐dialyzable and insoluble fraction. Alternatively, the reaction product was directly centrifuged to collect the insoluble fraction. Insoluble fractions were dried by washing with ethanol and acetone (OX‐CA). Zymosan A (ZYM) (Sigma‐Aldrich, St Louis, MO, USA) was prepared according to a previously described procedure. In brief, ZYM was suspended in endotoxin‐free water and autoclaved. All procedures were performed using endotoxin‐free injection water (Otsuka Pharmaceutical Co., Ltd, Tokyo, Japan). To prepare the water‐insoluble fraction, ZYM (100 mg) was washed with 10 mL of pyrogen‐free water and centrifuged, and the precipitate was collected. This procedure was repeated three times. The final precipitate was suspended in RPMI 1640 medium.

### Preparation of soluble fungal component

*Candida*‐soluble cell wall β‐d‐glucan (CSBG), which is a polysaccharide fraction, was prepared through DMSO extraction of NaClO‐oxidized *C. albicans* (NBRC 1385). Yeast cells (2 g) were suspended in 200 mL of 0.1 m NaOH and oxidized with an appropriate volume of NaClO solution at 4 °C for 1 day. After the reaction was completed, the reaction product was centrifuged to collect the insoluble fraction. Insoluble fractions were dried by washing with ethanol and acetone. Each dried fraction was suspended in DMSO, and the solubilized fraction was collected. The solubilized part was again precipitated with ethanol and acetone. The resulting material was designated as CSBG (*Candida* spp. β‐1,3‐/1,6‐d‐glucan) 23, 24, 25.

### Cell preparation

Bone marrow macrophages (BMMs) were isolated from C57BL/6 mice according to a standard procedure 26. The cells (1 × 10^6^·mL^−1^) were maintained in RPMI 1640 medium supplemented with 50 μg·mL^−1^ of gentamicin sulfate (Wako, Osaka, Japan), 10% (v/v) heat‐inactivated fetal bovine serum (Biosera, Kansas City, MO, USA), and 10% (v/v) L929‐cell conditioned medium [a source of macrophage colony stimulating factor (M‐CSF)], and the medium was changed every other day during the culture. The BMMs were used 7 days after isolation and culture. BMMs were cultured at 37 °C in a humidified atmosphere of 5% CO_2_ and 95% air.

### Cytotoxicity assay

The cytotoxic effect of HALD on BMMs was determined according to a previously reported method with slight modifications 27. In brief, HALD was added to BMMs (1.75 × 10^5^ cells/well) cultured in a 96‐well flat bottom plate (Sumitomo Bakelite Co., Ltd, Tokyo, Japan). After the addition of HALD or SDS (0–100 μg·mL^−1^) for 2 days at 37 °C, the BMMs were washed twice with fresh RPMI 1640 medium and cultured in 10% FBS‐RPMI medium containing 0.5 mg·mL^−1^ of 3‐(4,5‐dimethylthiazol‐2‐yl)‐2,5‐diphenyl‐tetrazolium bromide (MTT; dissolved in Dulbecco's phosphate‐buffered saline and filtered through a 0.2‐μm membrane) at 37 °C. After 4 h, the intracellular formazan crystals were dissolved in 200 μL of DMSO, and the absorbance values were measured at 550 nm. The absorbance values are expressed as the cell viability rate (%), with the rate of the control group as 100%.

### Measurement of cytokines

Bone marrow macrophages seeded in a 96‐well flat bottom plate (2 × 10^5^ cells/well) were pre‐cultured with granulocyte–macrophage colony‐stimulating factor (GM‐CSF) (1 ng·mL^−1^). After 24 h, HALD was added (0–50 μg·mL^−1^). After 24 h of incubation with HALD, BMMs were stimulated with OX‐CA, ZYM (50 μg·mL^−1^) or phorbol myristate acetate and ionomycin (PMA/i) (100 nm/500 nm) and cultured for 24 h. The culture supernatant obtained from BMMs stimulated with the fungal components was used for a cytokine assay. The concentration of cytokines in the supernatant was determined using TNF‐α ELISA MAX kit (BioLegend, San Diego, CA, USA), interleukin (IL) ‐6 ELISA MAX kit (BioLegend) and BD OptEIA mouse IL‐1β ELISA set (BD Biosciences, San Diego, CA, USA).

### Phagocytosis assay

Bone marrow macrophages (1.75 × 10^5^ cells/well) seeded in a 96‐well flat glass bottom plate (PerkinElmer, Inc., Waltham, MA, USA) were pre‐incubated with HALD (0–50 μg·mL^−1^). After 24 h, FITC‐labeled OX‐CA, ZYM, or latex (500 μg·mL^−1^) was added, and the BMMs were cultured for another 24 h. After culture, the supernatant was removed, and the cells were fixed with 10% paraformaldehyde (Nakarai Tesque Co., Ltd, Kyoto, Japan) for 15 min. After the removal of paraformaldehyde, the cells were incubated with PBS containing 2 μg·mL^−1^ 4′,6‐diamidino‐2‐phenylindole (Santa Cruz Biotechnology, Dallas, TX, USA) and a 500‐fold dilution of CellMask Deep Red Plasma Membrane Stain (Thermo Fisher Scientific, Waltham, MA, USA) at room temperature for 15 min in the dark. The plate was washed twice and sealed with 200 μL of PBS. Subsequently, the plate was scanned, and images were obtained with the Operetta High‐Content Imaging System (PerkinElmer) using a ×40 objective lens with 32 fields of view per well. The images were then analyzed with harmony software (PerkinElmer). The uptake of the fungal components was determined based on the intensity of FITC in the cytoplasm of BMMs.

### Measurement of reactive oxygen species

Bone marrow macrophages were cultured in 96‐well flat bottom microwell plates (Thermo Fisher Scientific) at 37 °C for 24 h in a humidified atmosphere of 5% CO_2_ and 95% air. The cells were maintained at 1.75 × 10^5^ cells/well in 10% FBS‐RPMI medium with GM‐CSF (1 ng·mL^−1^). Then, the cells were co‐cultured with HALD (2 μg·mL^−1^) in 0.1 mL of 10% FBS‐RPMI medium for 24 h. Horseradish peroxidase (400 μg·mL^−1^) (Wako) and luminol (1.72 mg·mL^−1^) (Sigma‐Aldrich) were dissolved in 10% FBS‐RPMI medium, and the luminol mixture was added to the culture plate. Subsequently, OX‐CA, ZYM (40 μg·mL^−1^) or PMA/i (200 nm/1 μm) was added to the culture plate. ROS levels were determined using a Microplate Luminometer LB96V (Berthold Technologies GmbH & Co. KG, Bad Wildbad, Germany).

### Binding assay of HALD to dectin‐1

A 96‐well plate (half area; Greiner, Tokyo, Japan) was coated with the CSBG (25 μg·mL^−1^) or HALD (0–100 μg·mL^−1^) preparation in 0.1 m carbonate buffer (pH 9.8) by incubating at 4 °C overnight. The plate was washed with PBS containing 0.05% Tween 20 (PBST; Wako Pure Chemical Co.) and blocked with PBST containing 0.1% BSA at 37 °C for 60 min. After the wash, biotin‐conjugated mouse dectin‐1 was added to the plate, and CSBG (25 μg·mL^−1^) was added with soluble antigen laminarin (Sigma‐Aldrich) or HALD (0–100 μg·mL^−1^) at 37 °C for 60 min. The plate was then washed with PBST, treated with peroxidase‐conjugated streptavidin PBST containing 0.1% BSA, and developed with a 3,3′,5,5′‐tetramethylbenzidine substrate system (KPL Inc., Milford, CT, USA). Color development was stopped with 1 m phosphoric acid, and absorbance was measured at 450 nm with 630 nm as the reference wavelength.

### Statistical analysis

Data are expressed as the mean ± standard deviation (*n *=* *3). At least three independent experiments were performed. Significant difference tests (Student's *t*‐test) were conducted between no‐treatment control and the HALD treatment group at each concentration. The significance of the differences between the means was assessed by Student's *t*‐test (*P <* *0.05; **P <* *0.01; ***P <* *0.001).

## Results

### *In vitro* polymerization of HA and LD by laccase

**Figure 1:**
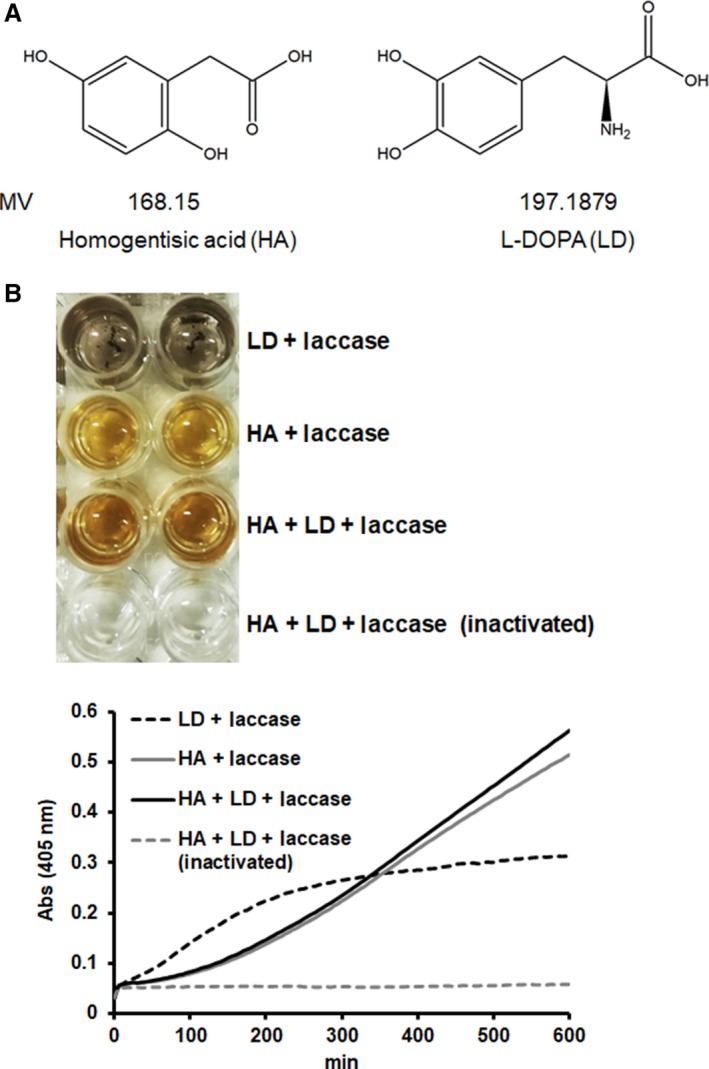
Solubilized melanin, synthesized by polymerization of HA and LD. (A) Structural formulae and molecular masses of HA and LD. (B) Polymerization of HA and LD was monitored by the change in absorbance at 405 nm. HA (0.19 mg·mL^−1^) and/or LD (0.19 mg·mL^−1^) was incubated at room temperature in 100 mm sodium acetate buffer (pH 5.0) containing 42.5 μg·mL^−1^ laccase.

The synthesis of solubilized melanin by laccase was examined at 37 °C. An HA, LD, or HA–LD mixture was used as a precursor (Fig. 1A). As shown in Fig. 1B, solubilized melanin was synthesized by allowing laccase to act on the solution containing the HA–LD mixture. The change in the color of this solution, observed as browning (increase of absorbance), was dependent on the reaction time. In contrast, color development did not occur when heat‐inactivated laccase was used. These results suggested that solubilized melanin is synthesized by the activity of laccase on the solution containing the HA–LD mixture. As the melanin consisting only of LD precipitated, the increase in absorbance ceased.

### Synthesis of the solubilized melanin

**Figure 2:**
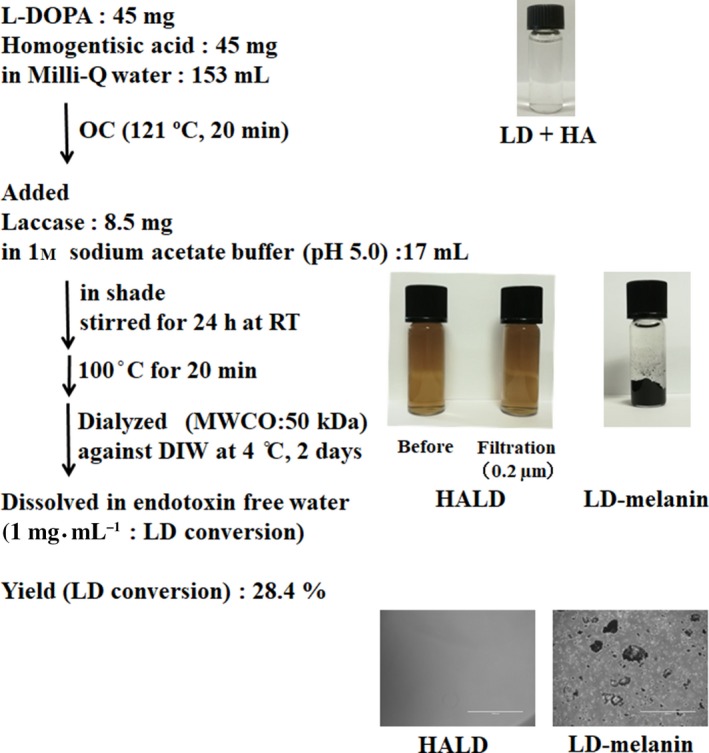
Preparation of the solubilized melanin. LD was mixed and polymerized with HA, and the polymerization reaction was carried out using laccase as a reaction catalyst. The yield was determined using the Folin–Ciocalteu method in terms of LD equivalents. Filtration was performed using a 0.2 μm membrane filter to confirm solubility. MMCO, molecular mass cut‐off.

To obtain the solubilized melanin, a polymerization reaction was carried out by mixing LD with an equivalent amount of HA, using laccase as a catalyst. In the polymerization reaction with LD alone, the mixture was completely separated into two layers, and particles were observed by optical microscopy, demonstrating that melanin was insoluble. On the other hand, HALD was not separated from the solvent, and no particles were observed. There was no change in the color tone of the solution containing HALD before and after filtration (0.2 μm membrane filter), and no particulate matter was observed in the filter (Fig. 2). The results indicated that the solubilized melanin was obtained by mixing HA and LD. The yield was 28.4% as calculated using the Folin–Ciocalteu method (adapted for LD). As shown in Table 1, the nitrogen content of LD‐melanin and LD was similar. The LD1 molecule was found to be the basic unit of LD‐melanin. Thus, the nitrogen of LD in melanin was suggested as the nitrogen source. The experimental nitrogen content value of HALD was close to the theoretical value of HA + LD (weight ratio 1 : 1). These results suggested that HA and LD polymerize at a ratio of 1 : 1.

**Table 1:** Elemental analysis of samples

	Element (%)		
	Carbon	Hydrogen	Nitrogen
Theoretical			
LD	54.82	5.62	7.10
HA	57.14	4.80	0.00
HA + LD	55.79	5.27	4.11
Experimental			
LD	54.60	5.62	7.10
HA	57.35	4.72	0.20
LD‐melanin	48.35	4.25	6.27
HALD	53.17	3.56	4.52

### Evaluation of the cytotoxic effect of the melanin on BMMs

**Figure 3:**
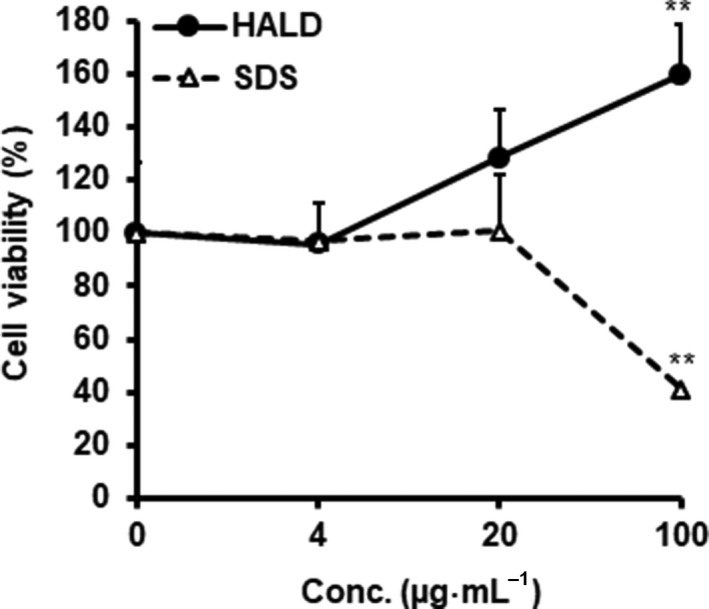
Cytotoxic effect of HALD, a synthetic solubilized melanin, on BMMs. BMMs were cultured for 24 h with HALD or SDS (0, 4, 20, or 100 μg·mL^−1^). After washing with RPMI medium, the supernatant was replaced with 10% FBS‐RPMI medium containing MTT and cultured for 4 h. After incubation, the supernatant was replaced with DMSO, and the absorbance was measured at 550 nm. The values shown are the mean ± standard deviation, *n* = 3. Significant differences from control: **P < 0.01. The significance of the differences between the means was assessed using Student's *t*‐test.

To evaluate the cytotoxicity of HALD, mouse BMMs were incubated with HALD or SDS, and the number of surviving BMMs was measured by the MTT method. As shown in Fig. 3, SDS decreased the viability of BMMs at 100 μg·mL^−1^ and higher concentrations. On the other hand, HALD was not cytotoxic at concentrations up to 100 μg·mL^−1^ and even induced the proliferation of the BMMs.

### Effect of HALD on cytokine production when stimulated by fungal components

**Figure 4:**
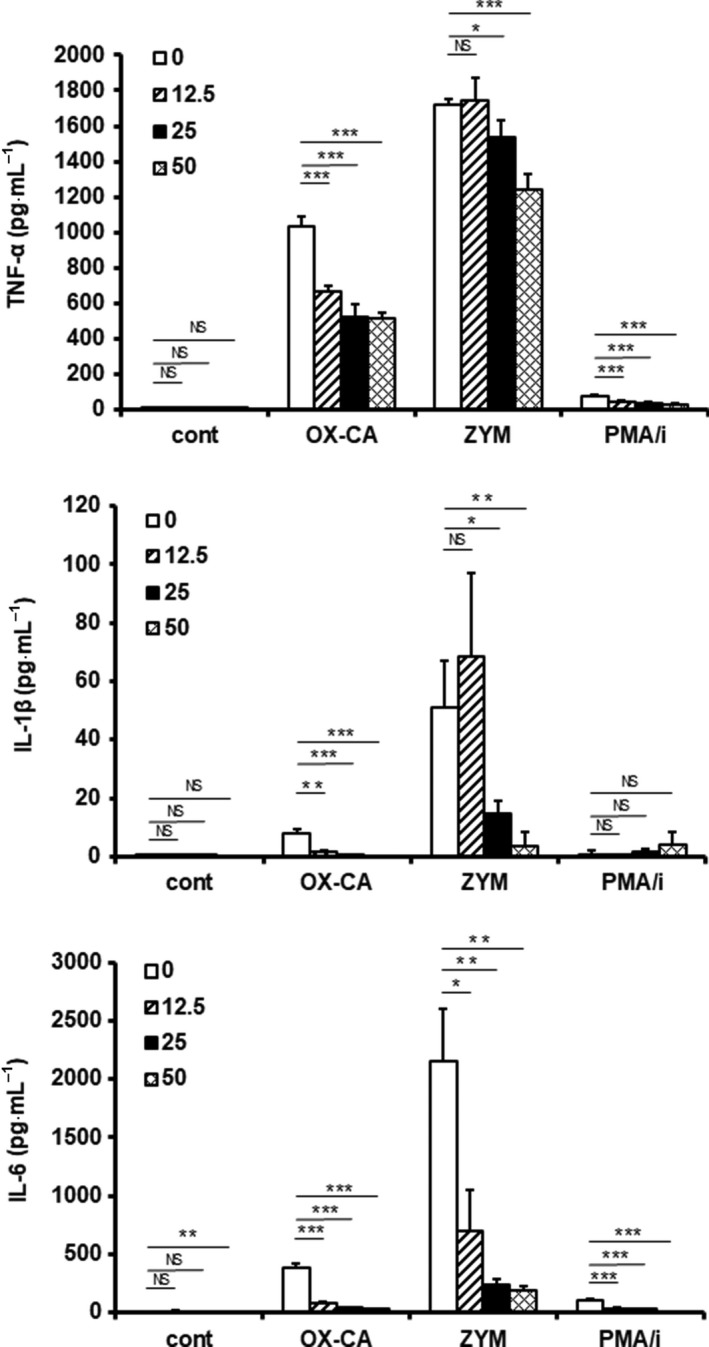
Inhibitory effect of HALD, a synthetic solubilized melanin, on TNF‐α production when stimulated by fungal components. BMMs were cultured for 24 h with HALD (0, 12.5, 25, or 50 μg·mL^−1^). After the co‐culture, particulate fungal components were added. After 24 h, the supernatant was collected, and the concentration of TNF‐α, IL‐1β and IL‐6 in the supernatant was determined by ELISA. The values shown are the mean ± standard deviation, *n* = 3. Significant differences from control: *P < 0.05; **P < 0.01; ***P < 0.001. The significance of the differences between the means was assessed using Student's *t*‐test.

Melanin‐producing *Cryptococcus* has been reported to reduce the ability of the infected host to produce cytokines. Therefore, we investigated whether the synthesized HALD could suppress the production of cytokines such as TNF‐α, IL‐1β, and IL‐6. As shown in Fig. 4, HALD suppressed inflammatory cytokine production by BMMs under OX‐CA, ZYM, or PMA/i stimulation. HALD inhibited the production of TNF‐α from 12.5 μg·mL^−1^ of HALD under OX‐CA or PMA/i stimulation. On the other hand, HALD suppressed TNF‐α production at a HALD concentration higher than 25 μg·mL^−1^ under ZYM stimulation. Under unstimulated conditions, HALD did not inhibit spontaneous TNF‐α production by BMMs. Expression of IL‐1β was suppressed by HALD to an extent similar to that of TNF‐α. On the other hand, under PMA/i stimulation, HALD did not inhibit spontaneous IL‐1β expression by BMMs. Expression of IL‐6 by BMM was suppressed by HALD in all stimulation groups. Under unstimulated conditions, 50 μg·mL^−1^ of HALD inhibited spontaneous IL‐6 expression by BMM.

### Suppressive effect of HALD on the phagocytic system

**Figure 5:**
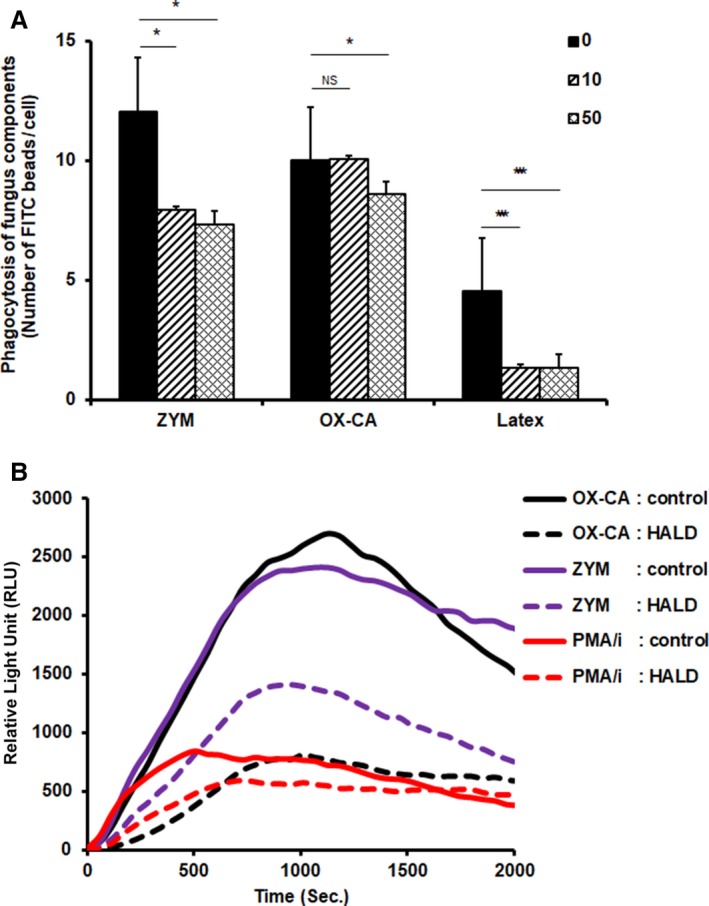
Effect of HALD, a synthetic solubilized melanin, on the phagocytic system. (A) BMMs were incubated for 24 h with HALD (0, 10, or 50 μg·mL^−1^). After culture, FITC‐labeled ZYM, OX‐CA, or latex was added to the culture plate, and the cells were cultured for another 24 h. After culture, the BMMs were removed from the supernatant and fixed with formalin. The nucleus and cytoplasm were stained with 4′,6‐diamidino‐2‐phenylindole and CellMask, respectively, to measure the phagocytosis of each FITC‐labeled sample. The average number of FITC puncta detected on each BMM is shown. The values shown are the mean ± standard deviation, *n* = 3. Significant differences from control: *P < 0.05; ***P < 0.001. The significance of the differences between the means was assessed using Student's *t*‐test. (B) BMMs were stimulated with GM‐CSF (1 ng·mL^−1^) for 24 h. Then, the BMMs were incubated with HALD (2 μg·mL^−1^). After 24 h, the luminol mixture was added to the culture plate. ZYM, OX‐CA, or PMA/i was added to each culture plate, and then, the chemiluminescence was measured immediately.

We investigated whether HALD could affect the phagocytic action of macrophages. FITC‐labeled particulate fungal components were added to BMMs treated with HALD, and the fungal components incorporated into BMMs were analyzed according to the particle number or FITC intensity. As shown in Fig. 5A, the BMM phagocytosis of OX‐CA, ZYM, and latex was significantly attenuated by HALD. The phagocytosis of ZYM and latex was suppressed by a low concentration of HALD. On the other hand, OX‐CA phagocytosis was suppressed by a high concentration of HALD. During the phagocytic process of particulate fungal components, degradation by ROS in macrophages is important to protect against infections. To evaluate the effect of HALD on ROS production, macrophages co‐cultured with HALD were stimulated with fungal components, and the amount of ROS produced was measured. As shown in Fig. 5B, HALD inhibited OX‐CA‐induced and ZYM‐induced ROS production. HALD also inhibited PMA/i‐induced ROS production. The inhibition of ROS production by HALD was weaker under PMA/i stimulation than under ZYM or OX‐CA stimulation.

### Binding of HALD to dectin‐1

**Figure 6:**
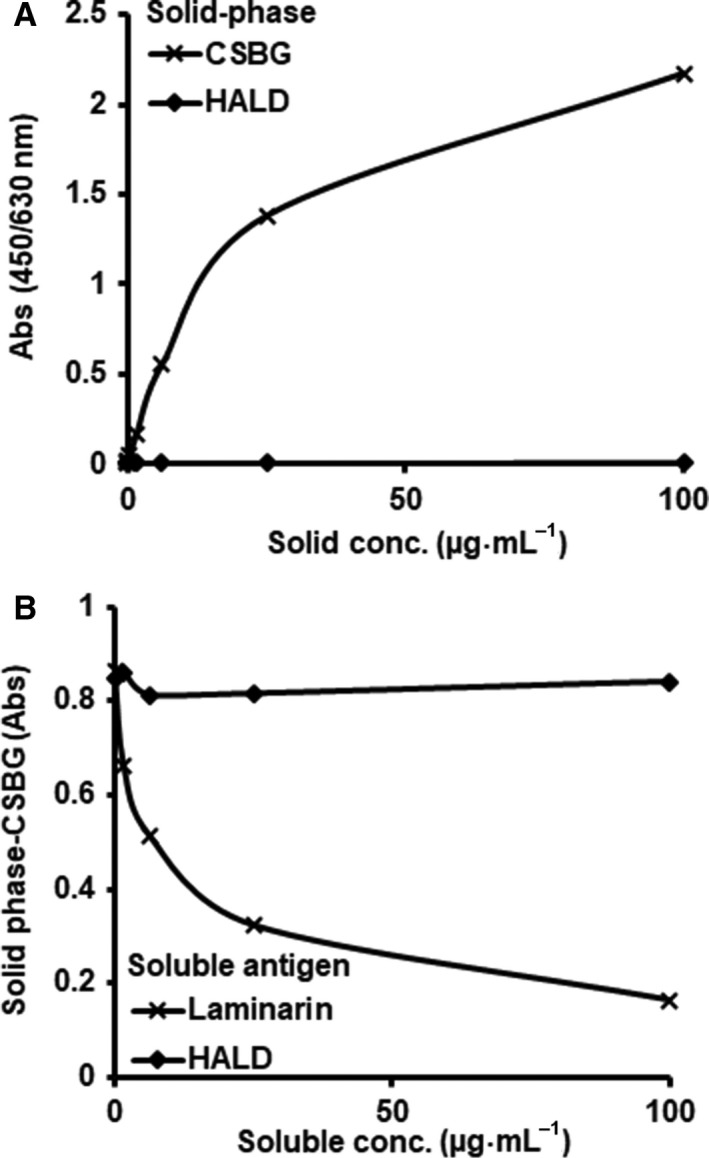
Reactivity of HALD, a synthetic solubilized melanin, to dectin‐1. (A) Reactivities of dectin‐1 on plates coated with CSBG or HALD. ELISA plates were coated with various concentrations of CSBG or HALD. Biotin‐conjugated dectin‐1 was added to each well precoated with CSBG or HALD, and the titer of antibody binding was detected with streptavidin–peroxidase. (B) Specificity and cross‐reactivity of dectin‐1 to CSBG via ELISA. Biotin‐conjugated dectin‐1 was added to each well in the presence of various concentrations of soluble antigens, laminarin, or HALD. Dectin‐1 that bound to the plate was detected with streptavidin–peroxidase.

In order to clarify the influence of HALD on the binding of β‐glucan to dectin‐1, we performed an ELISA. First, CSBG bound to dectin‐1, but HALD did not (Fig. 6A). Competitive ELISA was performed using laminarin or HALD as a soluble ligand for dectin‐1. Laminarin suppressed the binding of dectin‐1 to CSBG, but HALD did not (Fig. 6B).

## Discussion

Melanin is pigment that is synthesized by various organisms and is widely distributed in nature. The pathogenicity of pigments produced by microorganisms such as melanin has been revealed 4.

*Pseudomonas aeruginosa* and *Aeromonas* spp. produce pigment components. One type of pigment component is pyomelanin 8, 9. Pyomelanin is a water‐soluble polymer in which HA is synthesized by the catalysis of homogentisic acid 1,2‐dioxygenase 8. Pyomelanin acts defensively against antibacterial drugs and oxidative stress in *P. aeruginosa* 5.

Some fungi such as *Cryptococcus* and *Aspergillus* (conidia) produce melanin from LD and the like using laccase as a catalyst 6, 13, 28, 29. It has been reported that melanin‐producing fungi inhibit the host's immune system 30. This strongly suggests that melanin inhibits host immunity in fungal infection. However, melanin synthesized using tyrosine or LD as a precursor is completely insoluble in the solvent 16. Therefore, it makes analysis of the effect of melanin on the immune system *in vitro* difficult.

We focused on melanin‐producing fungi utilizing not only LD but also HA as a precursor of melanin synthesis 7. We predicted that HA could contribute to the solubilization of fungal melanin. To analyze the influence of melanin on the immune system *in vitro*, we attempted to synthesize soluble melanin. Commercially available laccase derived from *Trametes* spp. was used as a catalyst to polymerize equivalents (mass ratio) of LD and HA. The progress of the polymerization reaction was confirmed on the plate by the absorbance of the solution (Fig. 1). Polymerized reaction proceeded with laccase in both LD, HA and HA + LD. As LD became insoluble, the increase in absorbance saturated. On the other hand, the absorbance of HA and HA + LD continued to rise. Melanin polymerized only with LD became insoluble quickly and separated from the liquid phase. No particles were observed in HALD by microscopic analysis (Fig. 2). From these results, melanin (HALD) synthesized by mixing equal amounts of LD and HA was shown to be soluble. The results of the nitrogen content assessment suggested the ratio of HA and LD in solubilized melanin to be 1 : 1 (w/w) (Table 1). That fungal laccase catalyzes the oxidation reaction of HA has been reported 31. Therefore, it is considered that HALD was synthesized by a heterogeneous polymerization reaction of HA and LD catalyzed by fungal laccase. It was found that LD‐melanin is insoluble but can be solubilized by adding HA during the polymerization process. From the change in absorbance shown in Fig. 1, it was predicted that the polymer of HA was soluble and was a pyomedanine‐like pigment. Therefore, HA was suggested to contribute to the solubilization of HALD. It was suggested that HALD has a part of the structure of soluble melanin such as pyomelanin in part of the structure of fungal melanin derived from LD.

As melanin‐producing *Cryptococcus* has been reported to suppress the immune system, we analyzed the immunosuppressive effect of the synthesized HALD in this study. To confirm that the immunosuppressive effect of HALD was not cytotoxic, we evaluated the cytotoxicity of HALD by the MTT assay. When SDS was used as a positive control, there was a strong cytotoxic effect; however, HALD did not affect the proliferation of BMMs (Fig. 3). Therefore, HALD was not cytotoxic to BMMs, suggesting that any immunosuppressive effects would not result from cytotoxicity.

Analysis of the effect of HALD on cytokine production stimulated by particulate β‐glucan revealed that HALD suppressed the production of TNF‐α, IL‐1β and IL‐6 under the stimulation of fungal components (Fig. 4). HALD did not affect spontaneous cytokine production, and the intensity of the inhibitory effect was different for each stimulant. Therefore, HALD may attenuate or suppress specific signals responding to some stimuli.

Analysis of the effect of HALD on the phagocytic system, which is a defense mechanism of macrophages, showed that the phagocytosis of latex and ZYM was suppressed more strongly. On the other hand, the suppression of OX‐CA phagocytosis by HALD was relatively weak (Fig. 5A). Since latex does not have any epitopes on the bead surface, the suppression of signaling rather than the recognition of antigens may be involved in the inhibition of the phagocytic process. To analyze the effect of HALD in cells during the phagocytic process, the effect of HALD on ROS production under the stimulation of particulate fungal components was quantified. The production of ROS following the addition of ZYM or OX‐CA was strongly suppressed by HALD. On the other hand, the suppressive effect was weak under PMA/i stimulation (Fig. 5B). This was consistent with the findings of a previous study showing that ROS production induced by PMA/i was independent of phagocytosis 32. Under ZYM or OX‐CA stimulation, ROS was produced by phagocytosis, and HALD strongly suppressed ROS production. The results revealed the poor ability of HALD in inhibiting phagocytosis‐independent ROS production.

Low‐molecular‐mass β‐glucans, such as laminarin, have been reported to be substances that suppress the responsiveness of dectin‐1. The suppression of fungal components by low‐molecular‐mass β‐glucan is a result of a binding inhibition of β‐glucan to dectin‐1, which has been reported to be due to the antagonistic activity of dectin‐1 33, 34. However, it was shown that HALD did not inhibit the binding of β‐glucan to dectin‐1 (Fig. 6). This suggested that the inhibitory effect of HALD on fungal components was not dependent on dectin‐1 and that the inhibitory effect of HALD may have an effect on dectin‐1 via some signaling pathway.

In this study, we reported the effect of soluble melanin on the immune system, demonstrating that HALD suppressed the function of macrophages. Melanogenic *Cryptococcus* can suppress the inflammatory response and has been reported to suppress TNF‐α produced by macrophages and decrease the number of *Cryptococcus*‐specific lymphocytes 4. In addition, chromogenic strains can pass through the blood–brain barrier by surviving in macrophages via the Trojan‐horse mechanism, causing fatal central nervous system infections 11. These strains are highly invasive and exhibit enhanced resistance to phagocytic cells. The suppression of TNF‐α production, phagocytosis, and phagocytosis‐dependent ROS production is consistent with the characteristics of a melanin‐producing *Cryptococcus* and *Aspergillus* 4, 11, 13, 35, 36. In this study, we refer to *Aspergillus* and *Cryptococcus*, but we believe that the phenomena are common to fungal melanin. Overall, this study demonstrated the melanin‐mediated suppressive effect of melanin‐producing fungi on the host immune response.

The same strain of fungi produces various kinds of melanin pigments 37. Therefore, HALD is considered to be contained in a part of melanin produced by melanin‐producing fungi. Laccase is roughly classified into fungal laccase and laccase of higher plant in terms of substrate specificity 38. It has been reported that fungal laccase from each fungal species is identity in substrate specificity. Therefore, analyzing the influence of HALD on the immune system is considered to be an important approach for analyzing the influence of melanin produced by fungi on the host.

## Author contributions

KT designed the study and wrote the initial draft of the manuscript. NO contributed to analysis and interpretation of data, and assisted in the preparation of the manuscript. All other authors have contributed to data collection and interpretation, and critically reviewed the manuscript. All authors approved the final version of the manuscript and agree to be accountable for all aspects of the work in ensuring that questions related to the accuracy or integrity of any part of the work are appropriately investigated and resolved.

## Conflict of interest

The authors declare no conflict of interest.
